# Nitrogen and potassium interactions optimized asynchronous spikelet filling and increased grain yield of japonica rice

**DOI:** 10.7717/peerj.14710

**Published:** 2023-01-17

**Authors:** Weitao Xu, Jianming Li, Jiancheng Feng, Zhenzhu Shao, Yidan Huang, Wenfeng Hou, Qiang Gao

**Affiliations:** Jilin Provincial Key Laboratory of Soil Resource Sustainable Utilization for Commodity Grain Bases, Jilin Agricultural University/College of Resources and Environmental Science, Jilin Agricultural University, Changchun, China

**Keywords:** Asynchronous filling, Interaction effects of nitrogen and potassium, Japonica rice, Superior and inferior spikelets

## Abstract

Poor grain filling severely reduces rice yield. Fertilizers play a vital role in regulating grain filling, especially nitrogen (N) and potassium (K). In this field study we aimed to investigate the interactive effects of N and K on the asynchronous filling properties of superior and inferior spikelets of japonica rice. We looked at grain filling under three N rates (0, 90, and 180 kg N ha^−1^) and three K rates (0, 60, and 120 kg K_2_O ha^−1^) during 2020 and 2021. Across two years, the results showed that the combined use of N and K on superior and inferior spikelets significantly increased their weight by 1.29 mg and 2.31 mg, their maximum grain filling rate by 0.24 mg d^−1^ and 0.07 mg d^−1^, and their average grain filling rate by 0.21 mg d^−1^ and 0.06 mg d^−1^, respectively, in comparison with the control (N0K0) treatment. Likewise, K supply increased the average contribution rate of superior and inferior spikelets to yield by 9.1% and 10.0%, respectively. Correlation analysis showed that the grain filling rate of superior and inferior spikelets was an important factor in determining the spikelet weight, whereas the grain filling time was not. We also found that the 1,000-grain weight mainly increased after increasing the spikelets’ maximum grain filling rate and average grain filling rate. Collectively, these results illustrate that the combined use of N and K can optimize the asynchronous filling of superior and inferior spikelets and, in particular, enhance inferior spikelet weight with higher rice yield.

## Introduction

Rice (*Oryza sativa* L.) is a vital cereal crop that provides food for 50% of the world’s population (Muthayya et al., 2014). Rice accounts for a third of China’s total grain output, and its high and stable yield is crucial to national food security (Huang & Zou, 2018). The rice yield level is directly determined by grain filling. The rate and degree of grain filling in rice spikelets differ largely depending on their positions on a panicle (Zhang et al., 2010). Superior spikelets, located on the top part of the panicle, generally flower early and usually exhibit higher grain filling rates and final grain weight. On the other hand, inferior spikelets flower late, usually have lower a grain filling rate and final grain weight, and are located on the bottom part of the panicle (Yang et al., 2006; Zhang et al., 2010). In today’s agricultural production system, poor grain filling is one of the main factors restricting rice yield improvement (Yang & Zhang, 2010). External factors, such as nutritional status, planting density, temperature, and moisture, limit the development of rice grains (Li et al., 2011; Chen et al., 2016; Ao et al., 2019; Fu et al., 2019).

Nitrogen (N) and potassium (K) are two important elements that affect crop yield. N is an essential “life element” during rice growth and development and plays a decisive role in rice yield formation (Fageria & Santos, 2015; Zhang et al., 2020). N input can significantly increase grain yield by promoting the number of spikelets per panicle (Zhang et al., 2013; Wu et al., 2021). According to previous research, the maximum grain filling rate, average grain filling rate, and final grain weight of inferior grains of diverse rice varieties increased with N usage (Jiang et al., 2016). K plays an important role in the carbon and N metabolism of crops (Hu et al., 2015). Propagation by photosynthesis after flowering provides 70% of the photoconductive compounds used to fill the rice grain (Pan et al., 2011). The supply of K affects the synthesis of chlorophyll, the structure of chloroplasts, and increases the rate of CO_2_ assimilation, which enhances photosynthesis (Hou et al., 2020). Additionally, K supply increases the yield profit by promoting the translocation of non-structural carbohydrates and reducing the incidence of disease (Zhang et al., 2019). Numerous studies have shown that insufficient K application hinders increases in crop yield (Das et al., 2019; Zhang et al., 2019; Hu et al., 2021). However, in agricultural production, K fertilizer is easily neglected by farmers due to its higher price and lower yield increase rate (Darilek et al., 2009; Hou et al., 2019a).

The combined use of N and K has been shown to improve rice yield (Hou et al., 2019a; Hou et al., 2019b; Hou et al., 2020; Ye et al., 2021). The reason for this is that the supply of N and K improved canopy characteristics, resulting in a lower light extinction coefficient (Ye et al., 2021). In addition, adequate K increases the activity of enzymes involved in N metabolism (Hou et al., 2019a). A lack of N and K results in an unbalanced distribution of N in leaves (reducing the absolute content of photosynthetic N), which limits leaf photosynthesis and photosynthetic N use efficiency (Hou et al., 2019b). Previous studies have explored the mechanism by which N and K interaction enhances rice yield in terms of canopy performance (Ye et al., 2021) and nutrient acquisition and utilization (Hou et al., 2019a). However, it is unclear if and how the interactive effects of N and K would affect asynchronous filling properties of superior and inferior grains. To determine the grain filling process of grains with shells, the logistic mathematical model was a better fit compared to the Richards mathematical model (Jiang et al., 2014). Therefore, we investigated the superior spikelet and inferior grain filling parameters of japonica rice grain under different N and K supply rates using the logistic mathematical model. In addition, we identified the relationships between grain yield and superior and inferior spikelet grain filling parameters. In this study, we expounded: (1) the response of asynchronous filling of superior and inferior spikelets to N and K; and (2) the main factors to improve rice yield based on grain filling parameters. The results of this study further elucidate the mechanism by which N and K interaction improves japonica rice yield.

## Materials & methods

### Experimental site

**Figure 1:**
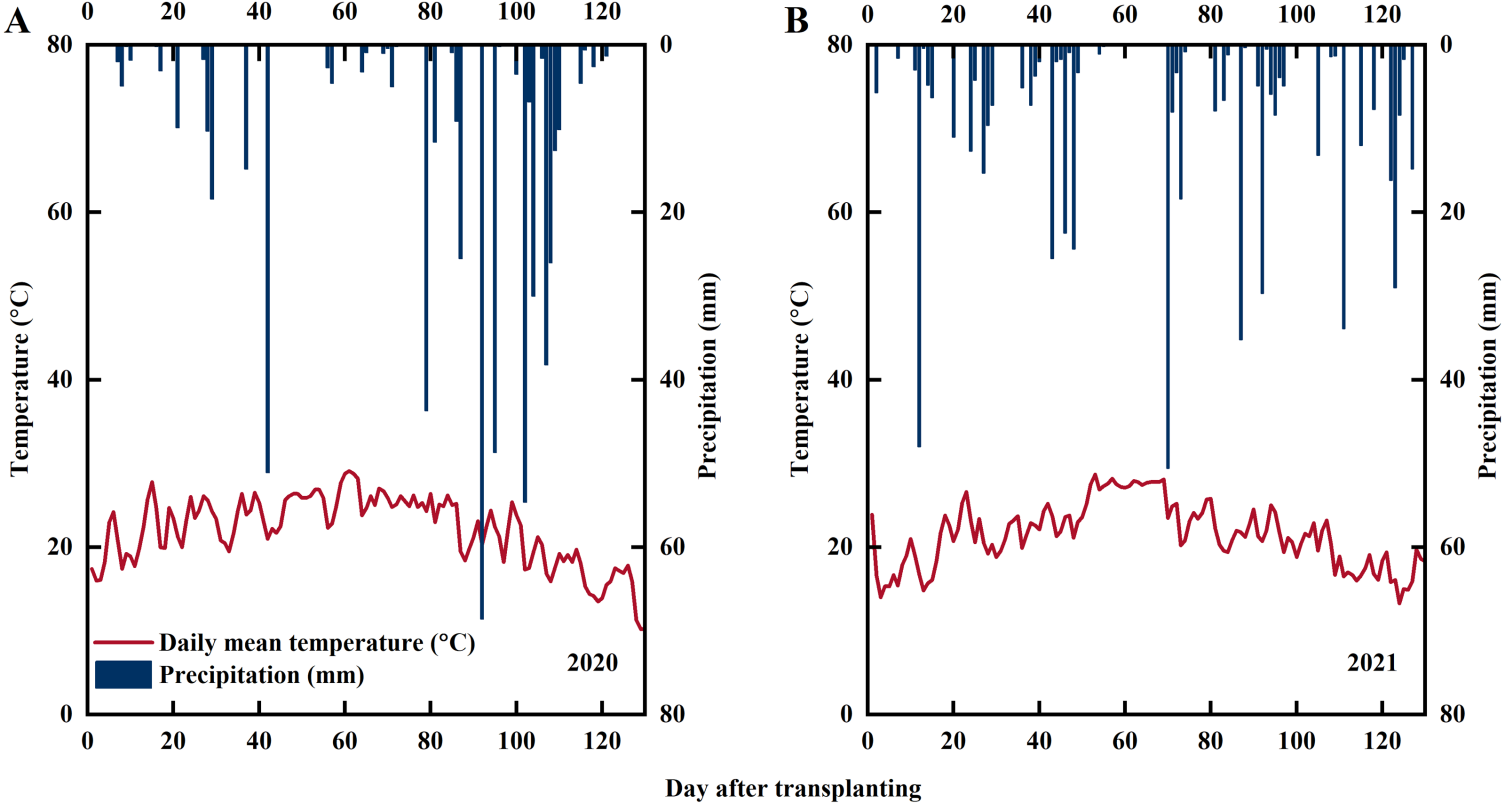
Statistics of precipitation and temperature during rice growing season in 2020 (A) and 2021 (B). Note: red line represents the average temperature, blue columns represents precipitation.

Experiments were conducted at Xihe Village, Lishu County, Jilin Province, China (43°43′N, 124°16′E) in 2020 and 2021 (the fieldwork was conducted on privately-owned land with the permission of the farmer, Jingfa Qu). The daily precipitation and average daily temperature during the experimental period are shown in Fig. 1. The soil used in the experiment was paddy soil. Soil samples from the plow layer (0–20 cm) were collected for soil analysis with the following characteristics: pH, 7.22; soil organic matter, 24.28 g kg^−1^; alkali-hydrolyzable N, 77.75 mg kg^−1^; Olsen-P, 20.34 mg kg^−1^; and NH_4_OAc-K, 84.67 mg kg^−1^. The rice variety used in this experiment was Jinongda 667, which is extensively cultivated in Jilin Province.

### Experimental design and field management

The experiment was comprised of a two-factor interaction between N and K fertilizer rates. Three supply rates were set for N (0, 90, and 180 kg N ha^−1^, abbreviated as N0, N90, and N180, respectively) and K (0, 60, and 120 kg K_2_O ha^−1^, abbreviated as K0, K60, and K120, respectively). There were a total of nine treatments for the interaction between N and K and each treatment was repeated three times. Phosphate fertilizer (90 kg P_2_O_5_ ha^−1^) was used for each treatment. Urea (46%), superphosphate (12% P_2_O_5_), and potassium chloride (60% K_2_O) were the sources of N, P_2_O_5_, and K_2_O. Fifty percent of N was applied as basal dressing one day before transplanting, 25% as the first topdressing at tillering stage, and the remaining 25% was used as the second topdressing at the panicle initiation stage. Seventy percent of K_2_O was applied as basal dressing one day before transplanting, while the remaining 30% was used as topdressing at the panicle initiation stage. The total P_2_O_5_ was applied as basal dressing one day before transplanting. Each plot was 20 m^2^ (4 × 5 m), set on a 0.6 m wide ridge, and covered with plastic wrap. The experiment was carried out by seedling transplanting, and seedlings were sown on April 2 and April 1 in 2020 and 2021, respectively. Transplanting was done by hand on May 25, 2020 and May 22, 2021 at a density of 22.7 × 10^4^ hills ha^−1^ with six to seven seedlings per hill, and harvested on October 1 and September 26 in 2020 and 2021, respectively. Pest, weed, and water management corresponded to the farmers’ conventional practices.

### Sampling and measurements

After flowering, one rice hill per plot was sampled at five-day intervals. The grains on the top three primary branches on the upper part of the panicle were defined as superior spikelets, while the grains on the three bottom secondary branches in the lower part were defined as inferior spikelets. Each part was removed and desiccated at 105 °C for 30 min and then dried at 75 °C to a constant weight. The grain filling process of superior spikelets and inferior spikelets were fitted using the logistic equation develooped by Jiang et al. (2014):

\begin{eqnarray*}W= \frac{A}{(1+B{e}^{-kt})} \end{eqnarray*}

where W is the grain weight (mg), A is the maximum grain weight (mg), t is the time after flowering (day), and B and k are coefficients determined by regression. The active grain filling period was defined as when W was between 5% (t_1_) and 95% (t_2_) of A. The average grain filling rate during this period was therefore calculated from t_1_ to t_2_.

The superior (inferior) spikelet contribution rate (%) was:

\begin{eqnarray*} \frac{\mathrm{Superior}~ \left( \mathrm{inferior} \right) ~\mathrm{spikelets~ weight}}{\mathrm{Grain~ weight}} \times 100\text{%}. \end{eqnarray*}

The grain yield of each plot was determined from 100 plants at physiological maturity and adjusted to 14% moisture content. Three emblematic hills were sampled from each plot at harvest to measure yield components. After the plant samples were dried and the rice ears were manually threshed and counted, we calculated the number of panicles, the number of spikelets per panicle, the filled grain rate, and the 1,000-grain weight.

### Statistical analysis

Analysis of variance (ANOVA) was performed using SPSS 25.0 software (SPSS Inc., Chicago, IL, USA). The least significant difference (LSD) test was used to evaluate differences among treatments, and the significance level was based on 5% probability level. The figures in this article were created using the Origin 2021 software program (Origin Lab, Northampton, MA, USA) and R v.4.1.0 software (R, Auckland, New Zealand).

## Results

### N and K interactions increased rice grain yield

The N and K rates and the interactions between them had significant effects on grain yield (Table 1). Both N and K had significant effects on rice yield, and there were significant interactions between the two elements. Compared to the N0 treatment, the rice yield of the N90 and N180 treatments increased by 51.2% and 102.6% in 2020, and 59.3% and 82.0% in 2021, respectively. Similarly, the rice yields under the K60 and K120 treatments increased by 5.5% and 5.0% in 2020, and 7.9% and 11.2% in 2021, respectively, in comparison with the K0 treatment (Table 1). The combined supply of N and K (N90K60, N90K120, N180K60, and N180K120) achieved higher grain yield than the single supply of only N or only K (N90K0, N180K0, N0K60, and N0K120). N supplied with K enhanced the rice yield by an average of 58.2% and 62.2% in 2020 and 2021, respectively, compared with the N0K0 treatment, and the maximum increase rates were 116.8% and 105.9%, respectively.

**Table 1:** Effects of N and K on rice yield and yield components.

	Treatment		Yield (kg ha^−1^)	Panicle (No. m^−2^)	Spikelets (No. panicle^−1^)	Filled grain rate (%)	1,000-grain weight (g)
2020	N0	K0	3555 bC	227 bC	75 bC	91.0 bB	20.8 bA
				K60	3673 aC	257 aC	77bC	91.9 abB	20.8 abB
			K120	3783 aC	265 aB	80 aC	92.3 aB	21.0 aC
		N90	K0	5237 bB	272 aB	85 bB	94.2 aA	20.9 bA
				K60	5699 aB	282 aB	97 aB	94.3 aA	21.1 abA
			K120	5717 aB	280 aB	100 aB	94.9 aA	21.3 aB
		N180	K0	7151 bA	335 bA	104 bA	95.2 aA	21.0 bA
				K60	7447 abA	381 aA	106 aA	95.8 aA	21.2 bA
			K120	7708 aA	388 aA	108 aA	95.8 aA	21.6 aA
		*ANOVA*						
		N		**	**	**	**	**
		K		*	**	**	**	**
	N × K		*	*	**	ns	ns
2021	N0	K0	3867 cC	177 bC	105 bB	90.9 aB	20.3 aB
				K60	4057 bC	194 abC	108 abC	91.4 aB	20.5 aB
			K120	4324 aC	204 aC	109 aC	92.1 aB	20.7 aB
		N90	K0	6290 aB	288 bB	113 bA	94.0 bA	20.3 bB
				K60	6490 aB	293 abB	116 abB	95.0 abA	21.0 aAB
			K120	6727 aB	308 aB	116 aB	95.3 aA	21.0 aA
		N180	K0	6934 cA	358 bA	114 cA	93.0 bA	20.7 bA
				K60	7400 bA	378 abA	122 bA	94.7 aA	21.0aA
			K120	7962 aA	391 aA	127 aA	95.5 aA	21.1 aA
		*ANOVA*						
		N		**	**	**	**	**
		K		**	**	**	**	**
	N × K		*	ns	**	ns	ns

**Notes.**

Note: Different lowercase (uppercase) letters indicate the significant differences between the different K (N) rates at the same N (K) level (*P* < 0.05). The variance analysis used two-way ANOVA (** *P* < 0.01, * *P* < 0.05, ns >0.05).

### N and K rate influenced yield components

Likewise, N and K rates significantly influenced the four yield components (the panicle per m^2^, spikelets per panicle, filled grain rate, and 1,000-grain weight). Additionally, there were significant interaction effects between N and K on the panicle per m^2^ and spikelet per panicle (Table 1). Compared with N0 treatment, the N supply enhanced the panicles per m^2^ and spikelets per panicle by 29.5% and 29.3% on average in 2020, and 75.2% and 10.0% on average in 2021, respectively. Similarly, compared with the K0 treatment, the panicles per m^2^ and spikelets per panicle increased by 11.0% and 7.8% on average in 2020, and 7.5% and 5.2% on average in 2021, respectively, with the K treatment. The combined use of N and K (N90K60, N90K120, N180K60, and N180K120) enhanced the panicles per m^2^ and spikelets per panicle by 71.1% and 43.6%, and 121.4% and 15.9% across two years, respectively, in comparison with the N0K0 treatment.

### N and K interactions exhibited greater grain filling rates and heavier final grain weight

**Figure 2:**
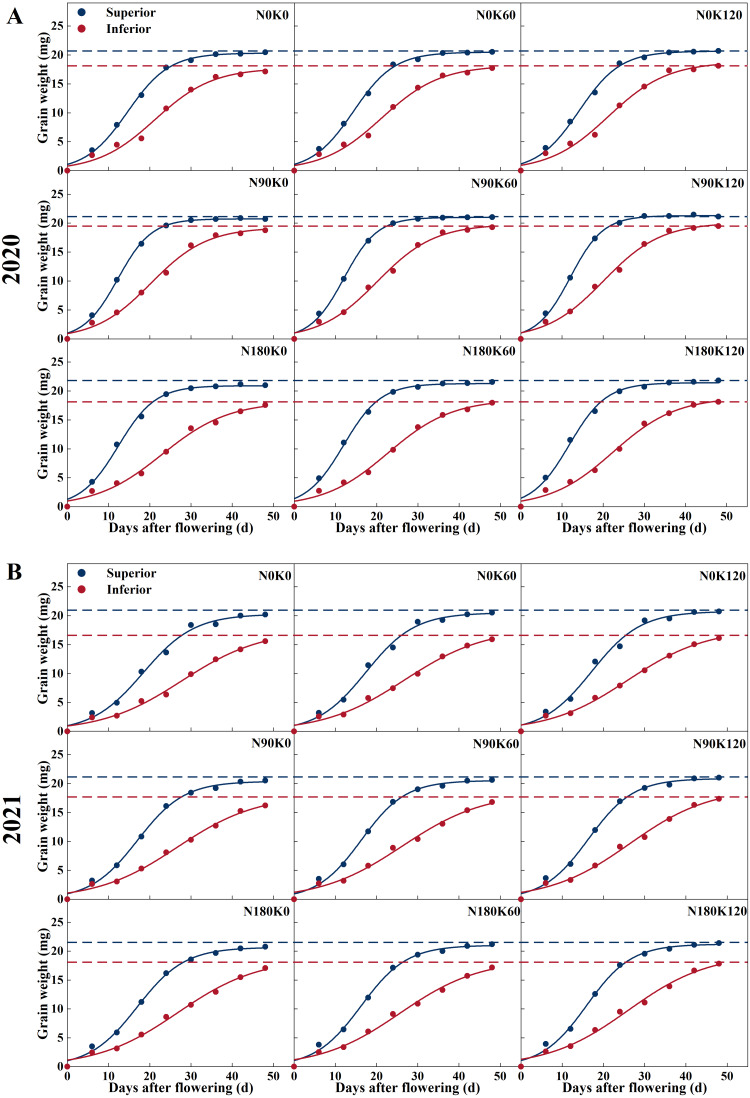
Grain filling curves of rice under different N and K treatments in 2020 (A) and 2021 (B). Note: blue curve represents the inferior spikelets, red curve represents the inferior spikelets.

**Figure 3:**
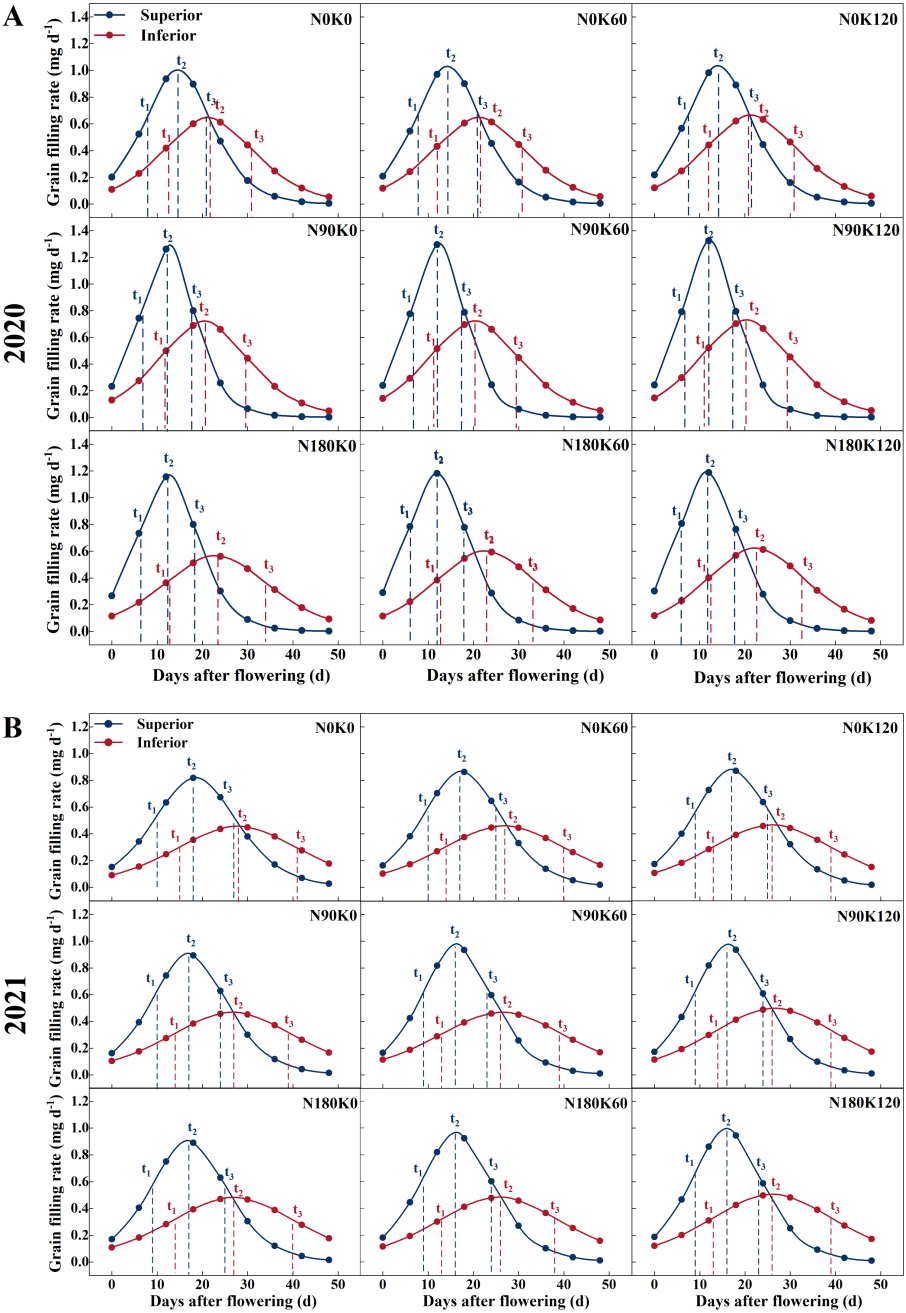
Grain filling rate curves of rice under different N and K treatments in 2020 (A) and 2021 (B). Note: blue curve represents the inferior spikelets, red curve represents the inferior spikelets, t_1_ represents the start of the rapid growth stage, t_2_ represents the maximum growth stage, t_3_ represents the end of the rapid growth stage.

The procession of grain filling of superior and inferior spikelets is illustrated in Figs. 2 and 3. With the advancement of grain filling days, the grain weight of superior and inferior spikelets progressively increased, showing a slow-fast-slow growth trend. Significant differences were found between superior and inferior spikelets during asynchronous filling. Superior spikelets from all treatments exhibited a greater grain filling rate and heavier final grain weight, which when compared with the inferior spikelets, was as expected.

N treatment without K (N90K0, N180K0) and K treatment without N (N0K60, N0K120) adversely affected grain filling. Under K supply conditions, superior and inferior spikelet weight was directly proportional to N supply rates. Compared with the N0 treatments, the N supply increased the superior and inferior spikelet weights by 0.64 mg and 0.87 mg on average in 2020, and 0.43 mg and 1.20 mg on average in 2021, respectively (Fig. 2). For each N level, superior and inferior spikelet weights continuously increased with K supply rates. Compared with the K0 treatments, the K supply increased the superior and inferior spikelets weight by 0.41 mg and 0.63 mg on average in 2020, and 0.42 mg and 0.58 mg on average in 2021, respectively. Compared with N0K0, the weight of the superior and inferior spikelets increased by 1.36 mg and 2.37 mg in 2020, and 1.21 mg and 2.24 mg in 2021 for the combined N and K supply (N90K60, N90K120, N180K60, and N180K120). The effect of fertilization on inferior spikelets was better than on superior spikelets.

Figure 3 shows that the superior spikelets entered the start of the rapid growth stage, maximum growth stage, and end of the rapid growth stage significantly earlier than the inferior spikelets. The three stages differed by 3–6 days, 6–11 days, and 10–17 days, respectively. Due to the grain filling rate of the inferior spikelets at each stage being significantly lower than that of the superior spikelets, the duration of the rapid growth stage was prolonged by 7–12 days.

### N and K supply optimized grain filling characteristic parameters

Table 2 summarizes the grain filling parameter values under different N and K combinations. Grain filling was well simulated by the logistic mathematical model: all R^2^ were >0.98. The maximum grain filling rate and average grain filling rate of the superior spikelets all increased with the N and K supply rate across two years. The supply of N increased the maximum grain filling rate and average grain filling rate of the superior spikelets by 0.22 mg d^−1^ and 0.19 mg d^−1^ on average in 2020, and 0.09 mg d^−1^ and 0.08 mg d^−1^ on average in 2021, respectively, compared to the N0 treatment (Table 2). Similarly, the supply of K increased the maximum grain filling rate and average grain filling rate of superior spikelets by 0.03 mg d^−1^ and 0.03 mg d^−1^ on average in 2020, and 0.06 mg d^−1^ and 0.05 mg d^−1^ on average in 2021, respectively, in comparison with the K0 treatment. Compared with the N0K0 treatment, the maximum grain filling rate and average grain filling rate of the superior spikelets increased by 0.32 mg d^−1^ and 0.28 mg d^−1^ in 2020, and 0.16 mg d^−1^ and 0.14 mg d^−1^ in 2021 for the combined use of N and K (N90K60, N90K120, N180K60, and N180K120).

**Table 2:** Logistic equations for superior and inferior grain filling.

Treatment		Equations		t_1_ (d)		t_2_ (d)		t_3_ (d)		△t(d)		Gm (mg d^−1^)		Ga (mg d^−1^)		R^2^	
		2020	2021	2020	2021	2020	2021	2020	2021	2020	2021	2020	2021	2020	2021	2020	2021
Superior spikelets	N0K0	*y* = 20.33/(1 +17.77e^−0.1969t^)	*y* = 20.27/(1 +19.51e^−0.1613t^)	8	10	15	18	21	27	13	16	1.00	0.82	0.88	0.72	0.9953	0.9903
		N0K60	*y* = 20.49/(1 +17.70e^−0.2003t^)	*y* = 20.50/(1 +19.04e^−0.1688t^)	9	10	15	17	22	25	13	16	1.03	0.87	0.90	0.76	0.9944	0.9909
		N0K120	*y* = 20.66/(1 +16.69e^−0.1989t^)	*y* = 20.75/(1 +18.09e^−0.1687t^)	8	9	14	17	21	25	13	16	1.03	0.87	0.90	0.77	0.9938	0.9884
		N90K0	*y* = 20.74/(1 +19.73e^−0.2437t^)	*y* = 20.40/(1 +20.19e^−0.1765t^)	7	10	12	17	18	24	11	15	1.26	0.90	1.11	0.79	0.9969	0.9950
		N90K60	*y* = 21.00/(1 +19.66e^−0.2471t^)	*y* = 20.53/(1 +21.01e^−0.1864t^)	7	9	12	16	17	23	11	14	1.30	0.96	1.14	0.84	0.9966	0.9947
		N90K120	*y* = 21.29/(1 +19.90e^−0.2489t^)	*y* = 20.88/(1 +20.12e^−0.1834t^)	7	9	12	16	17	24	11	14	1.33	0.96	1.16	0.84	0.9965	0.9940
		N180K0	*y* = 20.92(1 +15.35e^−0.2214t^)	*y* = 20.67/(1 +19.04e^−0.1738t^)	6	9	12	17	18	25	12	15	1.16	0.90	1.02	0.79	0.9932	0.9949
		N180K60	*y* = 21.29/(1 +14.26e^−0.2219t^)	*y* = 21.03/(1 +18.74e^−0.1803t^)	6	9	12	16	18	24	12	15	1.18	0.95	1.04	0.83	0.9931	0.9939
	N180K120	*y* = 21.43/(1 +13.69e^−0.2219t^)	*y* = 21.22/(1 +18.91e^−0.1846t^)	6	9	12	16	18	23	12	14	1.19	0.98	1.04	0.86	0.9917	0.9937
Inferior spikelets	N0K0	*y* = 17.74/(1 +21.25e^−0.1432t^)	*y* = 17.53/(1 +17.76e^−0.1035t^)	12	15	21	28	31	41	18	25	0.64	0.45	0.56	0.40	0.9853	0.9877
		N0K60	*y* = 18.19/(1 +19.55e^−0.1402t^)	*y* = 17.79/(1 +15.68e^−0.1027t^)	12	14	21	27	31	40	19	26	0.64	0.46	0.56	0.40	0.9882	0.9882
		N0K120	*y* = 18.80/(1 +19.44e^−0.1392t^)	*y* = 17.73/(1 +15.01e^−0.1045t^)	12	13	21	26	31	39	19	25	0.65	0.46	0.57	0.41	0.9860	0.9897
		N90K0	*y* = 19.26/(1 +19.90e^−0.147t^)	*y* = 18.03/(1 +15.73e^−0.1033t^)	11	14	20	27	29	39	18	26	0.71	0.47	0.62	0.41	0.9917	0.9897
		N90K60	*y* = 19.81/(1 +18.04e^−0.1437t^)	*y* = 18.46/(1 +14.18e^−0.1008t^)	11	13	20	26	29	39	18	26	0.71	0.46	0.62	0.41	0.9917	0.9856
		N90K120	*y* = 20.09/(1 +17.88e^−0.1433t^)	*y* = 19.26/(1 +15.16e^−0.1030t^)	11	14	20	26	29	39	18	26	0.72	0.50	0.63	0.43	0.9917	0.9874
		N180K0	*y* = 18.14/(1 +17.47e^−0.1242t^)	*y* = 18.75/(1 +15.58e^−0.1023t^)	12	14	23	27	34	40	21	26	0.56	0.48	0.49	0.42	0.9879	0.9895
		N180K60	*y* = 18.56/(1 +18.64e^−0.129t^)	*y* = 18.55/(1 +14.46e^−0.1039t^)	12	13	23	26	33	38	20	25	0.60	0.48	0.52	0.42	0.9908	0.9878
	N180K120	*y* = 18.93/(1 +18.77e^−0.1305t^)	*y* = 19.54/(1 +14.47e^−0.1027t^)	12	13	22	26	33	39	20	26	0.62	0.50	0.54	0.44	0.9903	0.9868

**Notes.**

Note: t_1_ represents the start of the rapid growth stage, t_2_ represents the maximum growth stage, t_3_ represents the end of the rapid growth stage, Δt represents the duration of the rapid growth stage, Gm represents the maximum grain filling rate, Ga represents the average grain filling rate.

In contrast to superior spikelets, inferior spikelets responded slightly differently to N treatment across the two years. In 2020, compared to the N0 treatment, the maximum grain filling rate and average grain filling rate of the inferior spikelets with the N90 treatment increased by 0.07 mg d^−1^ and 0.06 mg d^−1^ on average, but decreased with the N180 treatment by 0.05 mg d^−1^ and 0.04 mg d^−1^ on average (Fig. 3). Compared to the K0 treatment, the maximum grain filling rate and average grain filling rate of the inferior spikelets with the K treatment increased by 0.02 mg d^−1^ and 0.02 mg d^−1^ on average. In addition, the maximum grain filling rate and average grain filling rate of inferior spikelets under the N90K120 treatment were enhanced by 0.08 mg d^−1^ and 0.07 mg d^−1^, respectively, in comparison with the N0K0 treatment. In 2021, both N and K increased the maximum grain filling rate and average grain filling rate of the inferior spikelets. Compared with the N0 treatment, the supply of N increased the maximum grain filling rate and average grain filling rate of inferior spikelets by an average of 0.02 mg d^−1^ and 0.02 mg d^−1^, respectively. A similar effect was observed for K treatment, which increased the maximum grain filling rate and average grain filling rate of inferior spikelets by an average of 0.01 mg d^−1^ and 0.01 mg d^−1^, respectively. The 180 kg N ha^−1^ along with 120 kg K_2_O ha^−1^ resulted in a 0.05 mg d^−1^ and 0.04 mg d^−1^ increase in the maximum grain filling rate and average grain filling rate, respectively, of the inferior spikelets compared to that of the N0K0 treatment.

### N and K interactions increased superior and inferior spikelet contribution rates

In this experiment, the contribution rate of superior and inferior spikelets displayed the same change trend for two consecutive years (Table 3). Both N and K supply significantly affected the superior and inferior spikelet contribution rate in 2020 and 2021. N supply decreased the superior and inferior spikelet contribution rates by 6.2% and 5.9% on average in 2020, and 15.9% and 12.0% on average in 2020, respectively, in comparison with the N0 treatment (Table 3). Similarly, K supply increased the superior and inferior spikelet contribution rates by 9.6% and 9.7% on average in 2020, and by 8.6% and 10.3% on average in 2020, respectively, in comparison with the K0 treatment.

**Table 3:** The contribution rate of superior and inferior spikelets.

Treatment		Superior spikelets contribution rate (%)		Inferior spikelets contribution rate (%)	
		2020	2021	2020	2021
N0	K0	16.5 ± 0.4 bA	15.9 ± 0.7 bA	12.7 ± 0.1 bA	10.8 ± 0.7 bA
		K60	17.6 ± 1.0 abA	17.3 ± 0.3 aA	13.0 ± 0.6 bA	11.5 ± 0.4 abA
	K120	18.1 ± 0.8 aA	18.2 ± 0.5 aA	13.8 ± 0.3 aA	12.9 ± 1.1 aA
N90	K0	16.0 ± 0.5 bAB	14.0 ± 0.1 bB	11.6 ± 0.4 bB	10.0 ± 0.2 bAB
		K60	16.6 ± 0.0 abB	14.5 ± 0.3 bB	12.7 ± 0.7 aA	10.5 ± 0.2 aB
	K120	17.4 ± 1.0 aB	15.1 ± 0.3 aB	13.3 ± 0.3 aB	10.9 ± 0.2 aB
N180	K0	14.6 ± 1.0 bB	13.5 ± 0.2 bB	11.5 ± 0.5 bB	9.5 ± 0.4 bB
		K60	16.5 ± 0.4 aB	14.4 ± 0.6 abB	12.4 ± 0.4 aA	10.2 ± 0.3 aB
	K120	17.0 ± 0.8 aB	15.03 ± 0.6 aB	13.0 ± 0.1 aB	10.8 ± 0.2 aB
*ANOVA*					
N		**	**	**	**
K		**	**	**	**
N × K		ns	ns	ns	ns

**Notes.**

Note: Different lowercase (uppercase) letters indicate the significant differences between the different K (N) rates at the same N (K) level (*P* < 0.05). The variance analysis used two-way ANOVA (** *P* < 0.01, ns >0.05).

### The correlation between grain filling and yield in both superior and inferior spikelets varied in a day-dependent manner

**Figure 4:**
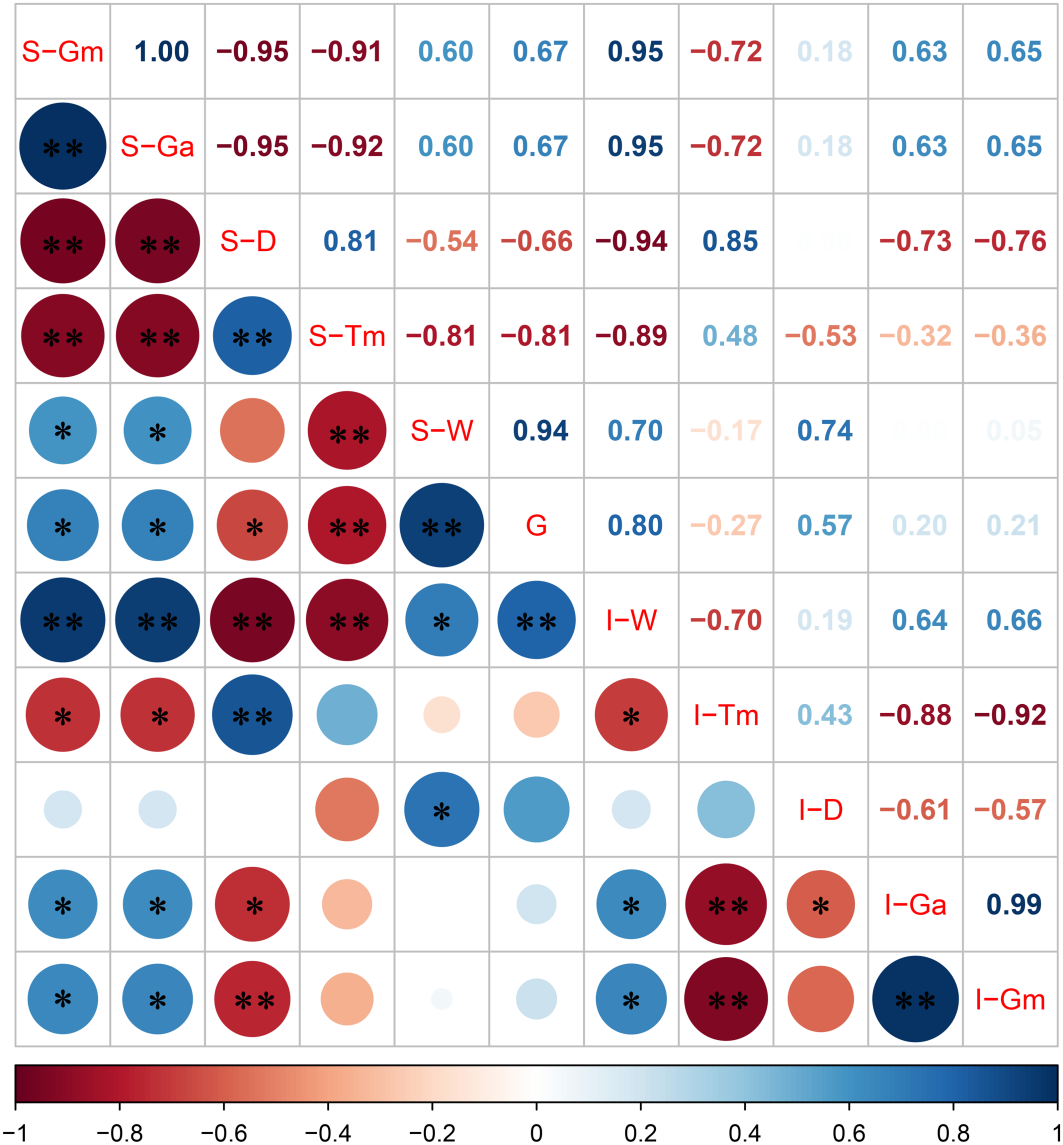
Correlation between grain filling characteristic parameters of superior and inferior spikelets with 1,000-grain weight. Note: blue and red represents positive and negative correlations, the size of the circle represents the strength of the correlation, S represents the superior spikelet, I represents the inferior spikelet, Gm represents the maximum growth rate, Ga represents the average growth rate, D represents the duration of the rapid growth stage, Tm represents the date when the maximum growth stage is reached, W represents the spikelet weight, G represents the 1,000-grain weight, ** represents statistical significance at *P* < 0.01, * represents statistical significance at 0.01 < *P* < 0.05.

The correlation between the grain filling characteristic parameters of superior and inferior spikelets with 1,000-grain weight is shown in Fig. 4. The Pearson correlation coefficient indicated that the 1,000-grain weight was positively correlated with S-Gm, S-Ga, I-Gm, I-Ga, and I-D, and the correlation coefficients were 0.67, 0.67, 0.21, 0.20, and 0.57, respectively (Fig. 4). Moreover, the grain weight and grain filling rate correlation at different stages after flowering were distinct (Table 4). Our analysis indicates that there was a significant positive correlation between grain weight and grain filling rate of superior spikelets 6 and 12 days after flowering and significant negative correlation between grain weight and grain filling rate of superior spikelets at 24, 30, 36, 42, and 48 days after flowering. In addition, the correlation between grain weight and grain filling rate of inferior spikelets was positively significant at 6, 12, and 24 days after flowering. These results indicated that the superior spikelets had a generally rapid filling rate within 12 days after flowering, while inferior spikelets needed more time (30 days).

## Discussion

### Effects of N and K on grain yield and yield components

Over recent decades, fertilizers have been widely investigated by scientists in order to increase rice yields (Gu et al., 2017; Peng et al., 2010). This study demonstrated the importance of balanced N and K levels for japonica rice in northeast China (Table 1). The two-year experiment results demonstrate that the interaction of N and K (N90K60, N90K120, N180K60, and N180K120) can further improve rice yield compared to a single supply of N (N90K0 and N180K0) or K (N0K60 and N0K120), which was consistent with previous studies (Hou et al., 2019a; Ye et al., 2021). The yield and N rate exhibited a parabolic relationship, where yield decreased when N exceeded a certain rate (Liu et al., 2019a; Liu et al., 2019b; Wang et al., 2019). Studies have shown that low levels of K limit the productivity of rice (Hou et al., 2020). Similarly with N fertilizer, an excessive supply of K will not further increase rice yields, but will instead only waste resources and money (Islam & Muttaleb, 2016; Ye et al., 2020). The grain yield did not decline in this experiment due to non-excessive N and K rates (Peng, Tang & Zou, 2009; Jin, 2012). On the other hand, K (N) with N (K) (N90K60, N90K120, N180K60, and N180K120) achieved higher grain yield than K (N) without N (K) (N0K60, N0K120, N90K0, and N180K0), implying that N (K) deficiency limited K (N) yield enhancement. Furthermore, the lower N supplying capacity of the soil (poor alkali-hydrolyzable N) in our experiment resulted in a greater yield of rice with N fertilizer compared to K fertilizer. We determined that N-K interaction improved yield by affecting panicle per m^2^ and the number of spikelets per panicle by analyzing the yield components (Table 1). The spikelets per panicle in 2020 was lower compared to those in 2021, possibly due to a short period of heavy rainfall during the flowering stage. The grain filling stage in 2021 had more rain and lack of sunshine compared with 2020, which caused lower filled grain rate and 1,000-grain weight.

**Table 4:** Correlations coefficient of the grain filling rate of superior grains and inferior spikelets with grain weight over two growth seasons.

	Spikelets position	Days after flowering (d)							
		6	12	18	24	30	36	42	48
Grain weight	Superior spikelets	0.900^**^	0.889^*^	−0.341	−0.974^*^	−0.919^*^	−0.860^*^	−0.857^*^	−0.747^*^
	Inferior spikelets	0.873^**^	0.844^*^	0.921	0.914^*^	0.051	−0.524	−0.140	0.058

**Notes.**

** represents statistical significance at *P* < 0.01, * represents statistical significance at *P* < 0.05.

In this study, the supply of N led to an increase in the filled grain rate and 1,000-grain weight, which differed from previous experiment results (Hou et al., 2019a; Ye et al., 2021), perhaps due to the practice of growing one crop of japonica rice per annum, the longer grain filling cycle, and the grain filling being more ample than in indica rice. K fertilization improved four yield components. A sufficient supply of K promoted root development, N metabolism-related enzyme activities, and the amount of non-structural carbohydrates in the stem, which may have increased the absorption of N nutrients, inherent buffering capacity of rice for grain filling, and the contribution to yield (Hou et al., 2019a; Zhang et al., 2019). In summary, in contrast to the sole supply of N or K (N90K0, N180K0, N0K60, and N0K120), the interaction of N and K (N90K60, N90K120, N180K60, and N180K120) was used to its full fertilizer potential in order to gain a higher yield.

### Effects of N and K on grain filling

The grain filling process is the foundation of shaping rice yield. The combined use of N and K could optimize the asynchronous filling properties of superior and inferior spikelets (Table 2). Our results in 2020 were similar to those of predecessors, where the maximum and average filling rate of inferior spikelets initially increased and then decreased with the increase of the N rate (Jiang et al., 2016). However, the results from 2021 showed that the maximum and average filling rate of inferior spikelets increased with the supply of N rate. The potential of the N180 treatment’s N fertilizer in 2021 to be better utilized was perhaps due to the slowdown of the duration of the rapid growth stage. K supply increased the maximum and average filling rate of superior and inferior spikelets, and optimized the grain filling of spikelets. Compared with 2020, the spikelets in 2021 had a longer duration of the rapid growth stage, but they also had a lower grain weight. The duration of the rapid growth stage of spikelets in 2021 was longer than that in 2020, which may be due to continuous rain during the grain filling stage and a lack of sunshine (Ren et al., 2003). This experimental phenomenon further confirms that the main factor affecting spikelet weight was the grain filling rate rather than the grain filling time (Fig. 4). Our study showed that N fertilization increased spikelet weight but decreased its contribution rate (Table 3), possibly due to the fact that the increase in sink capacity was greater than the weight of the spikelets with the supply of N rate. Sufficient N nutrient supply generally resulted in a higher sink capacity of rice (Kobayasi, Horie & Imaki, 2002; Fu et al., 2019). After N application, the weight of inferior spikelets increased more than that of the superior spikelets, resulting in the superior spikelet contribution rate declining more than the inferior spikelets’. In turn, K application adjusted the spikelet filling and improved the spikelet contribution rate (inferior spikelets outperformed superior spikelets), which was consistent with previous studies (Lei et al., 2015). The reason may be that K fertilizer influenced rice photosynthesis (Weng et al., 2007).

### Relationship between grain filling and yield

The grain filling stage is one of the important periods for the formation of rice yield, and the spikelet filling situation directly determined the final spikelets’ weight (Liu et al., 2019a; Liu et al., 2019b; Wei et al., 2017). Previous studies have shown that by increasing non-structural carbohydrates in stems and leaves at the heading stage of rice and optimizing the development of vascular bundles, the final weight of the inferior spikelets can be increased (Fu et al., 2011; Pan et al., 2011; You et al., 2021). N and K fertilizer could promote the accumulation and transport of non-structural carbohydrates and improve the rice 1,000-grain weight (Zheng et al., 2010). Ali et al. (2021) found a significant positive correlation between the filling rate of spikelets and the 1,000-grain weight. Our study demonstrated that the grain filling rates of superior and inferior spikelets were positively correlated with 1,000-grain weight (Fig. 4). Therefore, optimizing the grain filling rate of spikelets is an effective way to increase the 1,000-grain weight.

## Conclusion

This study showed that N and K interaction optimized the asynchronous filling properties of superior and inferior spikelets and increased the yield of japonica rice. The response of inferior spikelets to N and K was more sensitive than that of superior spikelets and the increase of the grain filling rate and grain weight of inferior spikelets was greater than that of the superior spikelets. As a result, the increased ratio of the contribution rate of inferior spikelets to yield was higher. By comparing data collected over two years, we found that higher grain weight could not be obtained by extending the grain filling period. Similarly, correlation analysis confirmed that the spikelet weight and 1,000-grain weight mainly increased by increasing the maximum grain filling rate and the average grain filling rate of spikelets. The interaction between N and K is a sustainable method of japonica rice production and provides a new theoretical basis for the high-yield and high-quality production of japonica rice from the perspective of superior and inferior spikelet filling properties.

##  Supplemental Information

10.7717/peerj.14710/supp-1 Data S1 Raw Data

## Additional Information and Declarations

### Field Study Permissions

The following information was supplied relating to field study approvals (*i.e.*, approving body and any reference numbers): The fieldwork was conducted in privately-owned land with the permission of the farmer, Jingfa Qu.

### Data Availability

The following information was supplied regarding data availability: The raw data are available in a Supplemental File.
